# CRISPR/Cas9 in Gastrointestinal Malignancies

**DOI:** 10.3389/fcell.2021.727217

**Published:** 2021-11-29

**Authors:** André Jefremow, Markus F. Neurath, Maximilian J. Waldner

**Affiliations:** Department of Medicine 1, Friedrich-Alexander-Universität Erlangen-Nürnberg, Erlangen, Germany

**Keywords:** gastrointestinal cancer, CRISPR/cas9, hepatocellular cancer, colorectal cancer, pancreatic cancer, esophageal cancer, cancer of the biliary tract

## Abstract

Gastrointestinal (GI) cancers such as colorectal cancer (CRC), gastric cancer (GC), esophageal cancer (EG), pancreatic duct adenocarcinoma (PDAC) or hepatocellular cancer (HCC) belong to the most commonly diagnosed types of cancer and are among the most frequent causes of cancer related death worldwide. Most types of GI cancer develop in a stepwise fashion with the occurrence of various driver mutations during tumor progression. Understanding the precise function of mutations driving GI cancer development has been regarded as a prerequisite for an improved clinical management of GI malignancies. During recent years, CRISPR/Cas9 has developed into a powerful tool for genome editing in cancer research by knocking in and knocking out even multiple genes at the same time. Within this review, we discuss recent applications for CRISPR/Cas9-based genome editing in GI cancer research including CRC, GC, EG, PDAC and HCC. These applications include functional studies of candidate genes in cancer cell lines or organoids *in vitro* as well as in murine cancer models *in vivo*, library screening for the identification of previously unknown driver mutations and even gene therapy of GI cancers.

## Introduction

Gastrointestinal (GI) malignancies are among the most frequently diagnosed tumors and therefore represent an enormous healthcare burden worldwide. Typical examples include colorectal cancer (CRC), pancreatic cancer, gastric cancer (GC), esophageal cancer (EC) or cancer of the liver and the biliary tract, most of which are among the 10 most frequent causes of cancer related death in males, females or both (Siegel et al., 2021). Classic treatment approaches consist of surgery, chemotherapy and radiation. Still, most tumors are detected in advanced stages of disease, so that patients frequently are in a palliative setting at diagnosis (Bray et al., 2018). During recent decades, intensive research on the pathophysiology of GI cancer resulted in the development of targeted therapies significantly improving the course of disease including reduced overall mortality and increased 5-years survival (Siegel et al., 2021). However, not all patients respond to current therapies and many patients develop resistance after an initial response. To enable earlier detection of gastrointestinal malignancies and improve therapeutic options, a better understanding of disease pathophysiology is required.

It is well established that tumorigenesis occurs through multiple steps and that these reflect genetic alterations, which are responsible for malignant transformation of normal cells (Hanahan and Weinberg, 2000). GI cancers include well-defined examples for the sequential accumulation of various oncogenic mutations during cancer development. For instance, Fearon and Vogelstein proposed a stepwise development of CRC from adenomas to carcinomas that is paralleled by mutations in specific genes (Vogelstein et al., 1988; Fearon and Vogelstein, 1990). These genes include adenomatous polyposis coli (APC), Kirsten rat sarcoma viral oncogene homolog (KRAS), phosphoinositide 3-kinase (PI3K), transforming growth factor β (TGF β) and others (Fearon and Vogelstein, 1990; Vogelstein et al., 2013). Until today, mutations in more than 140 genes that are involved in cancer development have been detected. These genes can either be described as oncogenes, where mutations lead to an activation of these genes, which then drive tumor growth, or tumor suppressor genes, where mutations lead to an inactivation of these so-called gate-keeper genes. To understand cancer pathophysiology, the precise evaluation of the function of tumor suppressor and oncogenes is of central importance. Common strategies to explore the function of individual genes *in vivo* include genetic engineering of mice using homologous recombination of embryonic stem cells including the generation of conditional alleles with site-specific recombinases such as Cre and flippase. However, these techniques are limited due to low gene targeting efficiency and high time requirement (Sanchez-Rivera and Jacks, 2015). Techniques that are more recent include the introduction of DNA double-strand breaks through non-homologous end joining pathway or the homology-directed repair with site-specific endonucleases such as zinc-finger nucleases or transcription activator-like effector nucleases (Barrangou and Doudna, 2016). However, these techniques are rather expensive and complex to design. In this regard, the discovery of clustered regularly interspaced short palindromic repeats (CRISPR) and CRISPR-associated protein 9 (Cas9) can be regarded as a breakthrough technique with the potential to revolutionize cancer research. After a short introduction into the CRISPR/Cas9 technology and its applications in cancer research, this review will give an overview on recent achievements based on the use of CRISPR/Cas9 in GI cancers.

## Genetic Engineering With Clustered Regularly Interspaced Short Palindromic Repeats/Cas9 and Its Applications in Cancer Research

**FIGURE 1:**
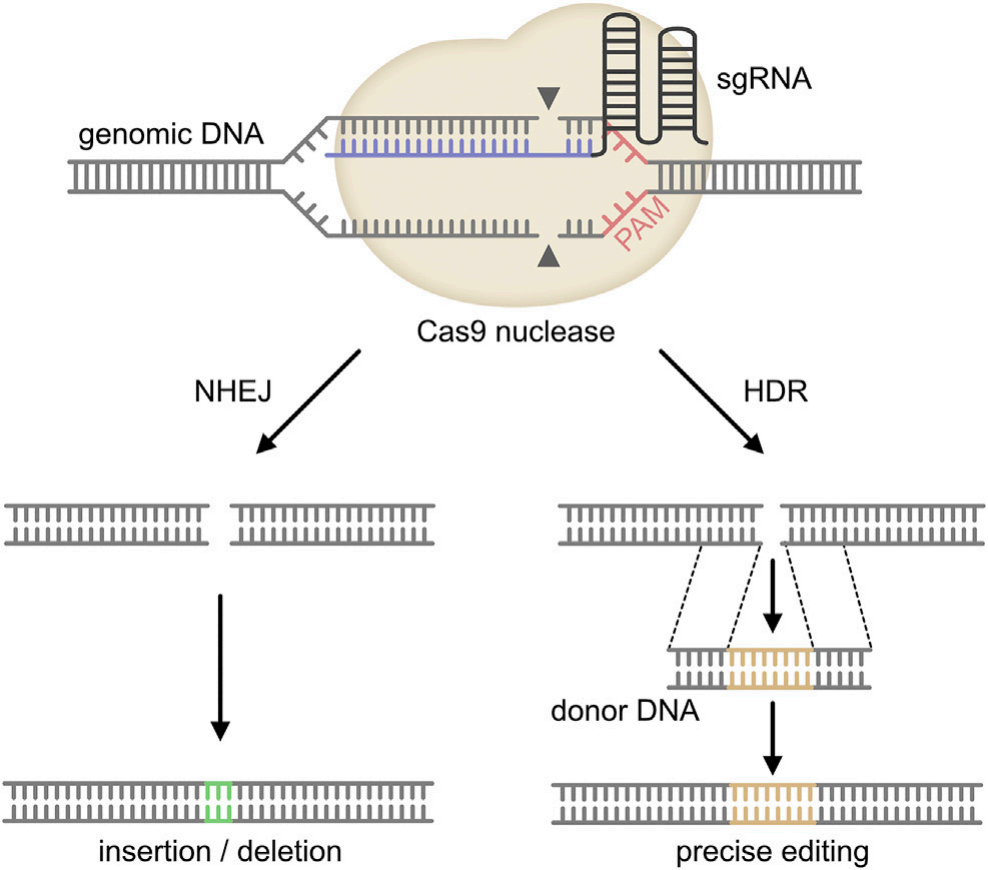
Mechanism of CRISPR/Cas9. The single guide (sg) RNA (target sequence in blue) is directed to the target site in the genomic DNA of the host. The Cas9 endonuclease forms a complex with the sgRNA and binds to the complementary DNA region upstream of the protospacer-adjacent motif (PAM). The Cas9 nuclease then cleaves both DNA strands about 3 base pairs upstream of the PAM. Repair of this DNA double-strand break either occurs through non-homologous end joining (NHEJ) or homology-directed repair (HDR). As NHEJ frequently induces insertions or deletions, this pathway results in a knock-out of the target gene. In contrast, HDR requires a donor DNA template, which will be inserted into the host DNA. Therefore, HDR can be used for knock-in applications (adopted from Moses et al., 2018 and Zhan et al., 2019).

In 1987, Ishino et al. described highly homologous sequences arranged as repeats with 32 nucleotides as spacing in *E. coli* for the first time (Ishino et al., 1987). These repeats with so far unknown function were later detected in many other bacteria and it was recognized that they are of extrachromosomal origin derived from foreign genetic material including bacteriophages (Bolotin et al., 2005; Mojica et al., 2005; Pourcel et al., 2005; Zhan et al., 2019). Subsequent research revealed that these clustered regularly interspersed short palindromic repeats (CRISPR) together with CRISPR-associated proteins (Cas) act as a bacterial defense mechanism against viral infection (Barrangou et al., 2007). CRISPR/Cas9 is activated and guided to a target site by a chimeric single guide RNA molecule (sgRNA), which contains the CRISPR targeting (crRNA) and the *trans*-activating RNA (tracrRNA) (Sanchez-Rivera and Jacks, 2015). For effective binding of the sgRNA to a target sequence, it must be close to a protospacer adjacent motif (PAM). Following binding to the target site and activation, CRISPR/Cas9 will induce double strand breaks (DSBs) in the target DNA. These DSBs are either repaired through the non-homologous end joining pathway (NHEJ pathway), which frequently results in sequence errors due to insertions or deletions, or the homology-directed repair (HDR) pathway, which results in precise repair in the presence of a exogenous donor DNA template and therefore can be used for knock-in strategies (see Figure 1) (Sanchez-Rivera and Jacks, 2015; Moses et al., 2018; Zhan et al., 2019). Nearly 10 years ago, first groups achieved targeted genetic engineering of mammalian cells using CRISPR/Cas9 (Doudna and Charpentier, 2014). Subsequently, CRISPR/Cas9 was continuously optimized to enable site specific deletion, insertions, knockouts, transcriptional activation or repression, epigenetic alterations or the generation of fusion proteins including linkage of fluorophores to targetproteins for imaging (Barrangou and Doudna, 2016).

Due to the simplicity and versatility of its usage, CRISPR/Cas9 was quickly applied to cancer research. Possible applications include the functional evaluation of individual genes in *ex vivo* or *in vivo* models, the discovery of cancer-relevant genes by CRISPR/Cas9 screens or cancer therapy via the introduction of new genetic material in cancer cells (Sanchez-Rivera and Jacks, 2015; Zhan et al., 2019). In the following sections recent achievements regarding these types of applications in the most common types of GI cancers will be discussed.

## Colorectal Cancer

Although various histologic types of colorectal tumors have been described such as lymphomas, neuroendocrine tumors, hamartomas etc.), the majority are carcinomas (Fleming et al., 2012). Among these, over 90 percent are adenocarcinomas, which develop through the adenoma-carcinoma sequence. As already described, Fearon and Vogelstein laid the foundation for the genetic basis of colorectal cancer with their groundbreaking work about the adenoma-carcinoma sequence (Vogelstein et al., 1988; Fearon and Vogelstein, 1990). The discovery of the tumor driving pathways WNT, KRAS, PI3K, TGFβ and TP53 not only enabled countless research activity, but has already changed the therapy of metastatic CRC (Karapetis et al., 2008).

However, the relative importance of individual mutations is still only poorly understood. This can be attributed to the fact that human CRC frequently contains 3-6 driver mutations (Vogelstein et al., 2013), and that previous methods did not allow a controlled deletion of so many candidate genes at the same time or sequentially for functional analysis of a combination of different mutations. This has been changed with the development of organoids, little organ-like tissues in a pars pro toto manner and their combination with CRISPR/Cas9 (Fujii et al., 2019). Organoids usually require various niche factors, e.g., Wnt, R-spondin, epidermal growth factor (EGF) and bone morphogenetic protein (BMP)/Tgfβ, and some inhibitors for survival and growth. Matano and coworkers were the first to perform sophisticated experiments using CRISPR/CAS9 technology in human intestinal organoids (Matano et al., 2015). They designed different organoids with a knockout of APC, TP53 and SMAD4 (SMAD family member 4) and a knock-in of KRAS and PIC3CA (further called AKSTP organoids). Strikingly, they could identify the success of the genetic manipulation by the fact, which niche factors were dispensable for organoid growth, e.g. APC deletion resulted in WNT-independent growth of organoids and organoids with mutations in APC, SMAD4, TP53, KRAS and PIK3CA were growing independently of niche factors. Drost et al. performed experiments with a similar approach (Drost et al., 2015). They made sequentially mutations in intestinal organoids using CRISPR/Cas9 starting with APC. Afterwards, they introduced mutations in TP53, KRAS and SMAD4. Like Matano et al., they confirmed successful transfection using culture medium lacking different substitutes. E.g., lack of nutlin-3 helped selecting intestinal organoids with TP53-deficiency. They also found that quadruple mutants can survive without niche factors (Drost et al., 2015). In both studies, CRISPR/Cas9 treated organoids were evaluated regarding *in vivo* growth capacities in murine models. Matano et al. transplanted their AKSTP organoids into kidney subcapsules of NOD-scid/IL2Rγnull mice. AKSTP organoids became visible tumors, while A organoids, which only harbor an APC mutation, could not form tumors. Of note, AKSTP organoids showed neither invasive growth nor metastatic ability in contrast to organoids derived from human CRC (Matano et al., 2015). Drost and his coworkers found that quadruple mutants (APC, TP53, KRAS, and SMAD4) showed invasive carcinoma growth and a bigger size in immunodeficient mice in comparison to triple mutants (APC, TP53, KRAS) that engrafted, but remained small (Drost et al., 2015). In fact, both publications were the first to show that CRISPR/Cas9 can be used to induce multiple mutations into human CRC organoids in a stepwise and controlled fashion and thereby can provide insights into the pathogenesis of CRC that were not possible before.

In addition to genetic engineering of organoids *ex vivo*, CRISPR/Cas9 can also be used to induce mutations in intestinal epithelial cells or organoids *in vivo* to study the effect of a sequential accumulation of various mutations on tumor growth. This has been shown by Roper et al., which used CRISPR/Cas9 to induce APC mutations in intestinal organoids of mice, which were orthotopically injected into the colon mucosa and subsequently edited for KRAS or TP53 mutations (Roper et al., 2017).

Besides the functional analysis of already known driver mutations, CRISPR/Cas9 is also increasingly being used within CRISPR screens for the identification and functional characterization of newer driver genes in CRC. For instance, You et al. compared the functional relevance of 19.050 human coding genes between KRAS mutated and wildtype HCT116 human CRC cells with CRISPR/Cas9 knockout screening (Yau et al., 2017). This approach enabled them to identify pathways such as nicotinamide adenine dinucleotide kinase (NADK) and fructose metabolism (ketohexokinase, KHK), which are specifically active in KRAS-mutated CRC, representing potential drug targets. Similar approaches have been used to identify genes that are involved in TGFβ-resistance (Michels et al., 2020; Ringel et al., 2020) or in the response to reactive oxygen species (Li et al., 2021).

In addition to functional studies on the pathogenesis of CRC, CRISPR/Cas9 has also been implicated as a potential tool for gene therapy. The feasibility of such an approach has been shown by Li et al., which corrected a mutation of the WNT pathway gene β-catenin in the human CRC cell line HCT116 with CRISPR/Cas9 (Li et al., 2020). In this study, β-catenin correction resulted in increased protein phosphorylation and reduced proliferation of CRC cells *in vitro* and also in a xenograft mouse model *in vivo*. These data clearly show the potential of CRISPR/Cas9 for therapeutic applications of CRC.

The studies described so far used CRISPR/cas9 to study molecular pathways involved in the adenoma-carcinoma sequence of CRC. However, CRC can also develop through serrated polyps, which are characterized by microsatellite instability (MSI) and frequent mutations of BRAF or DNA mismatch repair genes. Due to the genetic basis of the serrated pathway, CRISPR/Cas9 is also well suited for the functional evaluation of this type of CRC. For instance, Yan et al. evaluated genetic alterations of serrated polyposis families as well as sporadic serrated polyps and CRCs with various sequencing approaches (Yan et al., 2017). They detected a high frequency of RNF43 mutations and subsequently evaluated the function of RNF43, an E3 ubiquitin ligase, in organoids of serrated adenomas or mouse colon by inducing RNF43 mutations with CRISPR/Cas9. Organoids harboring RNF43 mutations showed reduced dependency on the growth factor R-spondin.

## Gastric Cancer

Gastric cancer (GC) includes gastric adenocarcinoma, lymphoma, sarcoma and carcinoid. Among these, adenocarcinoma is the most frequent one and all further discussion of GC will refer to adenocarcinoma. Similar to CRC, a stepwise development has also been described for gastric cancer (GC). For instance, current concepts propose that the sequential occurrence of chronic gastritis, atrophic gastritis and intestinal metaplasia are preceding the development of gastric cancer (Tan and Yeoh, 2015). These steps have also been associated with mutations in oncogenes such as KRAS, PIK3CA or tumor suppressor genes including TP53, AT-rich interactive domain-containing protein 1A (ARID1A), cadherin-1 (CDH1) etc.

With the development of gastric cancer organoids, CRISPR/Cas9 was also used to study the function of driver mutations in GC. For instance, Nanki et al. used CRISPR/Cas9 to establish a library for gastric cancer (Nanki et al., 2018). They investigated organoid lines from 37 human gastric cancers, which showed various niche factor dependencies. In these organoids, they identified a functional dependency of CDH1/TP53 mutations and R-spondin independency, which was successfully reproduced via CRISPR/Cas9 mediated knockout. E.g. they knocked down CDH1 and TP53 alone or in combination and found that only the combined mutation of both genes results in R-spondin independency. Beyond that, they demonstrated that CRISPR/Cas9 mediated activation of KRASG12V enabled gastric organoids to survive in an EGF and FGF10 (EF)-free culture medium (Nanki et al., 2018). In a similar approach, Lo and colleagues investigated the functional role of ARID1A in GC (Lo et al., 2021). They constructed gastric organoids with ARID1A-deficiency using CRISPR/Cas9. As organoids with ARID1A knockout were not able to survive, they generated a double knockout of ARID1A and TP53, leading to successful organoid survival with high-grade dysplasia features and improved engraftment in subcutaneous xenograft models. Zhang and his team investigated the relevance of Prostate-derived Ets factor (PDEF) in gastric cancer. They found that knocking PDEF out via CRISPR/Cas9 the AGS gastric cell line decreased proliferation, migration and invasion of the cells (Zhang Y.-Q. et al., 2019). In addition to organoids or cell lines, CRISPR/Cas9 has also been used to generate knock-out mice for the evaluation of gastric cancer in a study by Kim et al. (Kim et al., 2018).

Also in GC, CRISPR/Cas9 was used for screening of new and functionally relevant mutations driving tumor development. For instance, Chen and his team performed a kinome-wide CRISPR/CAS9 screening in gastric cancer cell lines with fibroblast growth factor receptor 2 (FGFR2) amplification to identify kinases that lead to a sensitivity for AZD4547, a FGFR inhibitor, since not every gastric cancer cell line with FGFR2 amplification is sensitive to it. One main finding was, that targeting Integrin-linked kinase with Cpd22 or Epidermal Growth Factor Receptor (EGFR) and human epidermal growth factor receptor 2 (HER-2) with lapatinib showed synergistic effects for the treatment of GC (Chen et al., 2019).

## Esophageal Cancer

Although esophageal cancer is among the less frequently diagnosed types of GI cancer, its incidence is continuously increasing. Most cases are either squamous cell carcinoma (SCC) or adenocarcinoma, which has now become the leading type of esophageal cancer in Western countries (Napier et al., 2014). Similar to other types of GI cancer, esophageal cancer is frequently diagnosed as late stage disease with only limited therapeutic options available. Thus, intensive research into the pathomechanisms of esophageal cancer has been pursued during recent years. This included also various applications of CRISPR/Cas9. For instance, Ji et al. studied the functional role of DEPTOR (DEP domain-containing mTOR interacting protein) in esophageal SCC using CISPR/Cas9 (Ji et al., 2016). Following the association of reduced levels of DEPTOR in tumor tissues with poor prognosis of esophageal SCC patients, the authors could show that DEPTOR negatively regulates SCC proliferation, migration and invasion by either overexpression or CRISPR-based deletion of DEPTOR in various SCC cell lines. Similarly, Zhai et al. studied the role of phospholipase C epsilon (PLCE1) in esophageal SCC (Zhai et al., 2017). Although various studies have shown that genetic variations of PLCE1 are associated with esophageal cancer susceptibility, the functional role of PLCE1 in esophageal cancer had not been evaluated before. Thus, the authors generated a PLCE1-deficient SCC cell line using CRISPR/Cas9, which showed reduced proliferation and invasion in comparison to a control cell line *in vitro* and *in vivo*. Similar studies have been performed to evaluate the functional role of MLL2, a histone methyltransferase, or PDHA1, the pyruvate dehydrogenase E1 alpha subunit, in SCC (Abudureheman et al., 2018; Liu et al., 2019). In addition to these studies on SCC, CRISPR/Cas9 has been also used to study the pathophysiology of esophageal adenocarcinoma (Zhang Q. et al., 2019).

Another application for CRISPR/Cas9 in SCC has been the evaluation of mechanisms involved in the response to anti-cancer therapy. For instance, Zhao et al. used a genome-scale CRISPR activation screening to identify mechanisms involved in the resistance against paclitaxel in SCC (Zhao et al., 2019).

Similarly to CRC, CRISPR/Cas9 has been also for gene therapy approaches. In one study, an integrated CRISPR interference (CRISPRi) system has been developed to selectively target an oncogenic isoform of TP63 in SCC both in cell lines *in vitro* and also xenograft mouse models *in vivo* (Yoshida et al., 2018).

## Pancreatic Cancer

Pancreatic cancer can arise from endocrine and exocrine cells and is differentiated based on histologic criteria. Among the different types of pancreatic cancer, pancreatic duct adenocarcinoma (PDAC) arising from exocrine cells accounts for over 90% of all cases (Grant et al., 2016). Similar to the other types of GI cancer discussed before, also PDAC arises from precursor lesions including pancreatic intraepithelial neoplasias (PanINs) and intraductal papillary mucinous neoplasms (IPMNs).

Driver mutations described in PDAC include mutations in the oncogene KRAS as well as mutations in the tumor suppressors CDKN2A, TP53 and SMAD4 (Grant et al., 2016). Also in the case of PDAC, CRISPR/Cas9 has been used in organoids to study the function of these mutations. An important example is a publication by Seino et al. (Seino et al., 2018). The authors made an organoid library from 39 pancreatic cancer patients. Among these, they identified three subtypes: a Wnt-non-producing subtype requiring Wnt from cancer-associated fibroblast, a Wnt-producing subtype and an R-spondin-independent subtype. Further experiments showed that Wnt-dependency is related to the expression of the transcription factor GATA6 (GATA6), as CRISPR/Cas9-based knockdown of GATA6 resulted in Wnt self-activation through upregulation of WNT7B in the previously Wnt-non-producing subtype (Seino et al., 2018). Armacki et al. showed, that Protein kinase D1 (PRKD1) is reduced in pancreatic tumors. Knockdown of PRDK1 with CRISPR/CAS9 in Panc1 cells was associated with an increased release of small extracellular vesicles, which promoted metastasis of xenograft and pancreatic tumors to the lung in mice *in vivo* (Armacki et al., 2020). Dong and colleagues used CRISPR/CAS9 technology to investigate the role of Hu-antigen R (HUR) that is highly expressed in pancreatic cancer. Cells with a HUR knockdown looked more like epithelium compared to control cells. They expressed more Claudin 1 and less Zinc finger protein SNAI1 (SNAIL) and Vimentin. Above that, knockdown of HUR via siRNA resulted in a reduction of cell migration. These data were further confirmed by inhibiting HUR with the specific inhibitor KH-3 in an orthotopic tumor model. In fact, also *in vivo* inhibition of HUR resulted in less cancer growth and metastasis (Dong et al., 2020).

Further studies on the use of CRISPR/Cas9 in pancreatic cancer have been summarized in a recent review by Yang et al. (Yang et al., 2019).

## Hepatocellular Cancer

With about 75–85% hepatocellular carcinoma (HCC) represents the most common primary liver cancer. Liver cirrhosis of any origin, chronic hepatitis B (HBV) and C (HCV) virus infections, non-alcoholic fatty liver disease (NFADL) and alpha-toxin poisoning depict the main risk factors. The Barcelona Clinic Liver Cancer (BCLC) algorithm stratifies HCC regarding number and size(s) of tumors, liver function and the performance status of the patient. Depending on BCLC, curative [surgical resection, radiofrequency ablation (RFA), liver transplantation (LTX)] or palliative [transarterial chemoembolization (TACE), systemic treatment or best supportive care (BSC)] therapy are applied (Jefremow et al., 2020). Research during the last years enabled new systemic therapies that are mainly based on immunotherapy and provide a significantly improved overall survival (OS) (Finn et al., 2020). Besides that, tyrosine kinase inhibitors like sorafenib (Llovet et al., 2008) or lenvatinib (Kudo et al., 2018) provide a backbone of systemic therapy (Jefremow et al., 2020). Key drivers of malignancy are e.g. TP53 mutations, telomere maintenance, the WNT/b-catenin pathway, and oxidative stress (Zucman-Rossi et al., 2015).

The SHARP trial in 2008 showed sorafenib as the first systemic agent to prolong OS (Llovet, et al., 2008). However, not all patients respond to sorafenib therapy and resistance is developed after initial therapeutic success. Consequently, first CIRSPR/CAS9 experiments in HCC dealt with the question, how to overcome sorafenib resistance or enhance sorafenib therapy. Gao et al. investigated the fibroblast growth factor 19 (FGF19)/fibroblast growth factor receptor 4 (FGFR4) axis. The knockdown of either FGF19 by shRNA or FGFR4 by CRISPR/CAS9 resulted in an increased sorafenib induced apoptosis in sorafenib-resistant HCC cell lines. Furthermore, the combination of sorafenib and ponatinib, which is used in chronic myeloid leukemia, showed similar results (Gao et al., 2017). Ardelt and his coworkers discovered that cyclin-dependent kinase 5 (Cdk5) can be crucial for sorafenib sensitivity in HCC. They inhibited Cdk5 by CRISPR/CAS9, short hairpin RNA (shRNA) or with specific inhibitors (roscovitine, dinaciblib, or LGR1407). The combination of Cdk5 sorafenib provided increased anti-tumor effects in comparison to sorafenib alone. They also showed that these *in vitro* results work *in vivo*. They administered sorafenib and a Cdk5 inhibitor to HCC xenograft mouse models and discovered a reduced growth of tumors (Ardelt et al., 2019). Sun et al. used the GeCKo library to identify Shugoshin 1 (SGOL1) as a marker for poor prognosis in HCC and a powerful inhibitor of sorafenib treatment. The mechanism, how SGOL1 knockout contributes to an increased sorafenib sensitivity remained unclear, as SGOL1 knockdown did not result in a greater amount of apoptotic HCC cells after sorafenib treatment in comparison to control cells (Sun et al., 2018). Wei and his coworkers underwent a similar approach, when they identified phosphoglycerate dehydrogenase (PHGDH) as crucial mediator of sorafenib resistance using the GeCKo library. Sorafenib induced PHGDH expression, which activated the serine synthesis pathway (SSP), an important driver pathway of sorafenib resistance. CRISPR/CAS9 mediated knockout of PHGDH sensitized HCC cell lines to sorafenib leading to decreased proliferation and increased apoptosis. Beyond that, the treatment with a PHGDH inhibitor, NCT-503, lead so similar results (Wei et al., 2019).

In addition to the evaluation of therapeutic resistance, various research groups used the CRISPR/CAS9 system to investigate novel tumor promoting genes. Iwagami et al. showed by CRISPR/CAS9 mediated knockout of Aspartate β-hydroxylase (ASPH) in the HepG2 hepatocellular carcinoma cell line leaded to less cell proliferation and induced senescence through p16 expression. The authors concluded that ASPH could be used for targeted therapy in HCC treatment. Treatment with MO-I-1151, an ASPH specific inhibitor, led to smaller tumors in comparison to a placebo group (Iwagami et al., 2016). Pott et al. found 34 phosphoproteins that were more expressed in HCC lysates than in non-HCC liver lysates. They could show that the total amount of Eukaryotic elongation factor 2 (eEF2) and its phosphorylated form are prognostic markers for HCC patients. Next, they took the CRISPR/CAS9 technology to knock out eEF2 out in human JHH5 hepatocellular cell lines leading to a decrease of proliferation and cell growth (Pott et al., 2017).

In contrast to normal liver tissue, which receives most nutrition and oxygen through the portal vein, HCCs are dependent on arterial perfusion. In this context, Bao and coworkers performed a very sophisticated experiment to investigate hypoxia in HCC. They used the GeCKo library in human MHCC97L cell lines and cultured the cells either under normoxia (20% O2) or hypoxia (1% O2) for 7 days. They found that protein-tyrosine phosphatase mitochondrial 1 (PTPMT1) was the third most important gene in hypoxia, right after the hypoxia inducible factors (HIF)-1α and 1β. PTPMT1 influences cardiolipin (CL) synthesis, which ensures electron transfer to mitochondria by building up electron transport chain (ETC). Strikingly, PTPMT1 knockout slowed proliferation and growth of HCC cells, especially in a hypoxic environment. Above that, a PTPMT1 inhibitor, alexidine dihydrochloride (AD), slowed cancer growth too. This happened again more effective under hypoxia than under normoxia (Bao et al., 2021). Furthermore, they used three different mouse models to investigate PTPMT1 *in vivo*: a hydrodynamic tail vein injection (HDTVi) model, where they injected a p53 KO by CRISPR-Cas9-KO plasmid and c-myc oe by sleeping beauty (SB) transposon system plasmid in C57BL/6N mice; a subcutaneous tumor model with nude mice using MHCC97L cells and orthotopic tumors with luciferase-labeled MHCC97L cells in nude mice. Treatment with a PTPMT1 inhibitor (AD) revealed that the tumors acquire more a more aggressive phenotype upon histology and that AD blocked lung metastasis in the orthotopic tumor model (Bao et al., 2021).

Engelholm and coworkers presented an important study regarding fibrolamellar hepatocellular carcinoma (FL-HCC). This type of HCC frequently develops in healthy livers of young people. Regarding the molecular basis of FL-HCC, a fusion of the DnaJ heat shock protein family (Hsp40) member B1 gene (DNAJB1) with the protein kinase cAMP-activated catalytic subunit alpha gene (PRKACA) has often been found in such tumors, but its cancer driving property had not been proven yet. Using the CIRSPR/CAS9 technology, Engelholm et al. generated a Dnajb1–Prkaca gene fusion in murine livers of 8-week-old female FVB/N mice through hydrodynamic tail vein injection of a control or Cas9 vector. Strikingly, 12 of 15 mice developed liver tumors with features of FL-HCC, while no control mouse showed a liver malignancy (Engelholm et al., 2017). This work is so important, because it provides the chance to investigate the rare FL-HCC *in vivo* and can contribute to develop specific therapies.

Although CRISPR/CAS9 technology plays its main part in preclinical research, scientists have taken first attempts to include it in the treatment of patients with liver cancer. Guo and his team performed the first studies with chimeric antigen receptor-redirected T (CAR T) cell therapy regarding HCC. They used CRISPR/CAS9 to disrupt programmed death 1 receptor (PD-1) on CAR T cells. They found a greater activity against PLC/PRF/5 HCC cells compared to wild-type CAR T cells. Above that, HCCs showed less growth in a xenograft model, where Guo et al. injected PLC/PRF/5 cells into NOD-scid-IL-2Rγ−/− (NSG) mice (Guo et al., 2018).

A challenge using CRISPR/CAS9 in treating gastrointestinal tumors remains: How should the plasmid not only come into the patient, but also to the right site? Wang and his coworkers used nanotechnology regarding this issue. They encapsulated Cas9-sgPlk-1 plasmids (CP) in several steps with lipid-Au-(LA) nanoparticles (LACP nanoparticles). LACP nanoparticles were able to enter tumor cells and release the plasmid in the cytoplasma after laser-triggered thermo-effects. TAT, a cell penetrating peptide guided the CP to the nucleus (Wang et al., 2018).

## Cancer of the Biliary Tract

With a 5-years survival of only 5–15%, cancer of the biliary tract including cholangiocarcinoma (CCC) or gallbladder cancer (GBC) is among the most aggressive types of cancer with only few therapeutic options available today (Lamarca et al., 2020). Due to the large heterogeneity of mutations involved in the pathogenesis of biliary tract cancer, genetically flexible models are urgently required to simulate human disease. Thus, CRISPR/Cas9 was also quickly applied to the study of biliary tract cancer. For instance, Erlangga et al. introduced previously described driver mutations of GBC together with p53-deletion into murine gallbladder organoids with CRISPR/Cas9 (Erlangga et al., 2019). These genetic modifications included mutant Kras and mutant ERBB2. Interestingly, both mutations led to GBC development, but with a different histological appearance. Of note, the developed model of GBC could be further used to study novel therapeutic approaches as for instance liposomal Irinotecan.

Similar to the other types of GI cancer, CRISPR/Cas9 has also been used to study the functional role of single genes in the development of biliary tract cancer. This includes studies on the role of the protein kinase CK2, TESC or ARID1A (Di Maira et al., 2019; Hsieh et al., 2020; Yoshino et al., 2020). Furthermore, CRISPR/Cas9 has been used to study mechanisms responsible for a response or resistance to anti-cancer therapeutics (Saha et al., 2016; Xu et al., 2019) or as a strategy for gene therapy (Carotenuto et al., 2020).

## Future Perspectives

Our review provides an overview about the different possibilities how CRISPR/Cas9 can be used for the investigation of GI malignancies for a summary of CRIPRS/Cas9 applications in GI cancer see Table 1. It was not our intention to present every single research aspect, but rather to show opportunities of the CRISPR/Cas9 technology. Whereas initial studies mainly used CRISPR/Cas9 for functional studies in organoids or cell lines of GI cancers *in vitro*, a second step involved *in vivo* studies using genetically modified organoids or even animals in various mouse models of GI cancer. Already until know, these strategies provided unique insights into the pathogenesis of GI cancers and especially disease heterogeneity. This has been further enhanced through CRISPR/Cas9 screening, which not only allowed the identification of new driver mutations in GI cancers, but also shed a light on genomic and functional differences between individual patients. However, several challenges need to be overcome before CRISPR/Cas9 can be translated for clinical routine management of GI cancers. For instance, off-target effects of CRISPR-based technologies are still a major concern, especially in the context of *in vivo* applications (Yang et al., 2021). Furthermore, current approaches only provide low efficiency in some cases and some genes cannot be targeted with standard approaches due to missing PAM sequences (Yang, et al., 2021; You et al., 2019). Intensive research is being performed to overcome these challenges and thus, CRISPR/Cas9 is still among the most promising candidates to become an indispensable tool for personalized medicine of GI cancers in the future.

**TABLE 1:** Applications of CRISPR/Cas9 in gastrointestinal cancers.

Type of application	Type of cancer	Cancer model	Gene	Ref.
Function of single genes	CRC	Human intestinal organoids, xenograft mouse models	APC, TP53, SMAD4, KRAS, PIC3CA	Matano et al. (2015)
	CRC	Human intestinal organoids, xenograft mouse models	APC, TP53, SMAD4, KRAS	Drost et al. (2015)
	CRC	Murine intestinale organoids, *in vivo* editing	APC, KRAS, TP53	Roper et al. (2017)
	GC	Human gastric organoids, xenograft mouse models	CDH1/TP53	Nanki et al. (2018)
	GC	Human gastric organoids, xenograft mouse models	ARID1A, TP53	Lo et al. (2021)
	GC	Human GC cell line	PDEF	Zhang et al. (2019a)
	EC	Human EC cell lines	DEPTOR	Ji et al. (2016)
	EC	Human EC cell line	PLCE1	Zhai et al. (2017)
	PDAC	Human PDAC organoids	GATA6	Seino et al. (2018)
	PDAC	Human PDAC cell line	PRKD1	Armacki et al. (2020)
	PDAC	Human PDAC cell line	HUR	Dong et al. (2020)
	HCC	Human HCC cell line	ASPH	Iwagami et al. (2016)
	HCC	Human HCC cell line	eEF2	Pott et al. (2017)
	BDC	Human gallbladder organoids	KRAS, ERBB2, TP53	Erlangga et al. (2019)
Screening for driver mutations	CRC	Human cell line	NADK, KHK	Yau et al. (2017)
	MSI CRC	Human intestinal organoids	RNF43	Yan et al. (2017)
	GC	Human GC cell line	FGFR2	Chen et al. (2019)
Response to therapy	HCC	Human HCC cell line	FGF19, FGFR4	Gao et al. (2017)
	HCC	Human HCC cell line, xenograft mouse models	CDK5	Ardelt et al. (2019)
	HCC	Human HCC cell line	SGOL1	Sun et al. (2018)
	HCC	Human HCC cell line	PHGDH	Wei et al. (2019)
Gene therapy	CRC	Human cell line	Β-catenin	Li et al. (2020)
	HCC	Human HCC cell line and CAR T cells, xenograft mouse model	PD1	Guo et al. (2018)

BDC: biliary duct cancer; CRC: colorectal cancer; EC: esophageal cancer; GC: gastric cancer; HCC: hepatocellular cancer; PDAC: pancreatic duct adenocarcinoma.
